# Helium-oxygen mixture: clinical applicability in an intensive care unit

**DOI:** 10.31744/einstein_journal/2018AO4199

**Published:** 2018-10-30

**Authors:** Milena Siciliano Nascimento, Érica Santos, Cristiane do Prado

**Affiliations:** 1 Hospital Israelita Albert Einstein, São Paulo, SP, Brazil.

**Keywords:** Respiratory insufficiency, Child, Intensive care units, neonatal, Oxygen inhalation therapy, Heliotherapy, Insuficiência respiratória, Criança, Unidades de terapia intensiva neonatal, Oxigenoterapia, Helioterapia

## Abstract

**Objective:**

To evaluate if distress respiratory decreases after using helium-oxygen mixture in pediatric patients diagnosed with bronchospasm.

**Methods:**

This is a retrospective, non-randomized study that included patients diagnosed with bronchospasm, who received a helium-oxygen mixture at three time points (30, 60, and 120 minutes) according to the organization protocol singular, and were admitted to the intensive care unit, from January 2012 to December 2013. This protocol includes patients with bronchospasm who sustained a modified Wood score of moderate to severe, even after one hour of conventional treatment.

**Results:**

Twenty children were included in the study. The mean score of severity of the disease at the initial moment was 5.6 (SD:2.0), and at moment 120 minutes, it was 3.4 (SD: 2.0). The severity score showed a significant improvement as of 30 minutes (p<0.001).

**Conclusion:**

The use of helium-oxygen mixture proved to be effective in diminishing the respiratory distress score for children with airway obstructions; it should be considered a supplementary therapeutic option, together with drug therapy, in specific clinical situations.

## RESUMO

**Objetivo:**

Avaliar se o desconforto diminui após o uso da mistura hélio-oxigênio em pacientes pediátricos com diagnóstico de broncoespasmo.

**Métodos:**

Estudo retrospectivo, não randomizado, no qual foram incluídos pacientes com diagnóstico de broncoespasmo que utilizaram a mistura hélio-oxigênio em três momentos (30, 60 e 120 minutos), seguindo o protocolo institucional, internados em unidade de terapia intensiva pediátrica de janeiro de 2012 a dezembro 2013. Este protocolo incluía pacientes com diagnóstico de broncoespasmo que mantivessem escore de Wood modificado de moderado a grave, mesmo após 1 hora de tratamento convencional.

**Resultados:**

Foram incluídas 20 crianças neste estudo. A média do escore de gravidade da doença no momento zero foi de 5,6 (DP:2,0) e, no momento 120 minutos, 3,4 (DP: 2,0). O escore de gravidade apresentou melhora significante a partir dos 30 minutos (p<0,001).

**Conclusão:**

A utilização da mistura hélio-oxigênio mostrou-se eficaz na redução do escore de desconforto respiratório de crianças com doenças obstrutivas e deve ser considerada recurso terapêutico complementar à terapia medicamentosa em situações clínicas específicas.

## INTRODUCTION

Helium gas was discovered by a French astronomer in 1868, but it was only isolated on Earth in 1895. Its molecular weight was determined in 1907.^(^ ^1^ ^)^ Its physical and chemical characteristics consist of low molecular weight, low density (one third of the density of oxygen), high viscosity, high diffusion coefficient (greater than that of carbon monoxide), and it is a biologically inert gas.^(^ ^1^ ^,^ ^2^ ^)^ Its use in clinical practice was based on these characteristics. Commercially, we find the mixture at concentrations 80:20 (80% helium and 20% oxygen), 70:30, and 60:40 – respectively, helium:oxygen concentrations.

The clinical applicability of Heliox^®^ is described primarily in obstructive diseases, such as asthma, bronchiolitis, and laryngitis.^(^ ^3^ ^-^ ^6^ ^)^ Due to the physical-chemical properties of the mixture (especially low density and high viscosity), it has a direct influence on the flow and resistance of the respiratory system.^(^ ^3^ ^,^ ^7^ ^)^ In 1934, Barach et al., confirmed that the use of the helium-oxygen mixture (heliox) decreases the transpulmonary pressure and improves tidal volume in an experimental study with tracheostomized dogs.^(^ ^8^ ^)^

Physiologically, the proximal airways have a turbulent flow pattern which becomes laminar in the terminal airways. In patients with obstructive disease, the turbulent flow of the proximal airways worsens respiratory distress and decreases lung ventilation. The use of heliox changes the flow pattern in the proximal airways from turbulent to laminar, thus decreasing respiratory work.^(^ ^9^ ^,^ ^10^ ^)^

The report of Heliox^®^ use in children with asthma has shown conflicting results. Conventional treatment is sufficient in most cases of disease exacerbation. Since it is a more expensive gas that oxygen, Heliox^®^ should not be used as a first choice; whereas in more severe cases, it seems to be beneficial, and its early use can decrease respiratory work and dyspnea.^(^ ^11^ ^)^

There is a great difference in establishing a consensus as to the use of Heliox^®^ in children due to the difference in criteria related to inclusion, time of intervention, treatment given, and severity of the disease in known studies. The role of Heliox^®^ in the exacerbation of asthma has not yet been determined.^(^ ^12^ ^,^ ^13^ ^)^

On the other hand, some studies have identified the effect of Heliox^®^ in the production of particles by jet nebulizers. The delivery efficacy of aerosol with Heliox^®^ is related to its low density. By becoming laminar, the turbulent flow improves ventilation in high resistance areas, affording optimization of aerosol deposition in lower airways.^(^ ^14^ ^-^ ^16^ ^)^

The use of the helium-oxygen mixture is not restricted to patients with spontaneous breathing. Its benefits may be associated to use of non-invasive and even invasive ventilation - as long as the synergistic treatments are taken into consideration.^(^ ^17^ ^-^ ^19^ ^)^

The effect of Heliox^®^ in decreasing the inflammatory process of respiratory distress syndrome patients, despite being confirmed, is not yet understood.^(^ ^20^ ^)^ Even so, the clinical applicability of the helium-oxygen mixture has gained prominence in intensive therapy environments, even though there still is no consensus as to its use.

## OBJECTIVE

To evaluate the efficacy of the clinical response to the use of the helium-oxygen mixture in patients with severe obstructive conditions.

## METHODS

A retrospective observational study on the analysis of the medical records of children, conducted in a private hospital in the city of São Paulo (SP).

Children aged zero to 14 years were included, admitted to the pediatric intensive care unit, with a diagnosis of bronchospasm and/or wheezing, and who used the helium-oxygen mixture, as per the organization protocol, between January 1^st^ , 2012 and December 31^st^, 2013.

The variables collected in the medical records for analysis of the clinical outcome were severity score; heart rate; respiratory rate; and oxygen saturation (SpO_2_) soon after the installation of the mixture, time point zero, 30 minutes, 60 minutes and 120 minutes.

Children were evaluated as to need for oxygen for maintenance of pulse oximetry (SpO_2_) greater than 92% and degree of respiratory distress, evaluated by the modified Wood clinical asthma score ( Table 1 ). Additional oxygen was initiated when SpO_2_ presented with values inferior to 92%. The oxygen flow utilized was the minimum necessary to maintain SpO_2_ >93%. The helium-oxygen mixture was initiated as per the organization protocol.

**Table 1:** Modified Wood clinical asthma score

	0	0.5	1	2
SatO_2_	≥95% in RA	90-94% in RA	≥90% with FiO_2_>21%	<90% with FiO_2_>21%
Breathing sounds	Normal	Slightly asymmetric	Very asymmetric	Diminished or absent
Wheezing	Absent	Mild	Moderate	Severe
Accessory muscle use	Absent	Mild	Moderate	Severe
Consciousness level	Normal	Agitated when stimulated	Depressed or agitated	Very depressed

Source: Translated and modified from: Cambonie G, Milési C, Fournier-Favre S, Counil F, Jaber S, Picaud JC, et al. Clinical effects of heliox administration for acute bronchiolitis in young infants. Chest. 2006;129(3):676-82.^(^ ^4^ ^)^

SatO_2:_ oxygen saturation; RA: room air; FiO_2 :_ inspired fraction of oxygen.

### Heliox® use protocol

The organization protocol for the use of the helium-oxygen mixture included patients diagnosed with bronchospasm and/or wheezing who maintained a Wood score ≥3 ( Table 1 ) after one hour of conventional treatment, regardless of the fraction of oxygen previously used.

The use of salbutamol, predinisolone, methylpredinisolone, inhalation with hypertonic solution, antibiotics, respiratory physical therapy, and oxygen therapy were considered conventional treatment, and was maintained even after initiating the helium-oxygen mixture.

**Figure 1:**
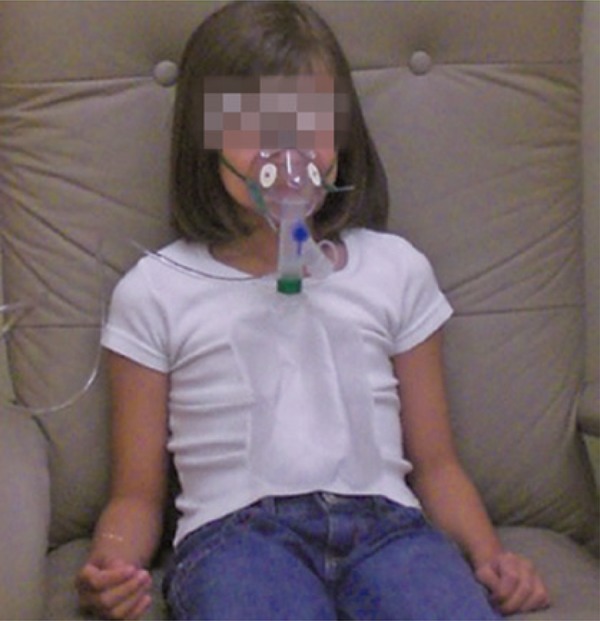
Mask specific for use with helium-oxygen mixture

For installation of the helium-oxygen mixture, the Heliox^®^ torpedo was used at the concentration of 70:30 (70% helium and 30% oxygen) with a specific non-rebreather mask ( Figure 1 ) and a flow of 10L/minute. Patients who did not maintain SpO_2_ >92% with 30% O_2_ had the mixture enriched with 2LO_2_, totalizing 40% de oxygen.

Patients who did not maintain SpO_2_ >92% with 40% oxygen and/or who did not present with any improvement in the severity score after one hour of Heliox^®^, had its use discontinued.

### Sample calculation

Sample calculation was based on the study by Cambonie et al.,^(^ ^4^ ^)^ who assumed a mean of respiratory distress score of 5.4 and standard deviation of 0.2. Thus, it was estimated that a sample of ten individuals was sufficient to detect a minimum difference of 0.3 unit in the distress score, hence assuming a type I error at 5% and a 90% power.

### Data analysis

Measurements were described by individual and average profile graphs, accompanied by confidence intervals. Assessment of the progression of the Wood clinical asthma score, heart rate, and respiratory rate was made by means of Student’s *t* test for paired samples.

The project was submitted to and approved by the Research Ethics Committee of *Hospital Israelita Albert Einstein* , official opinion number 687.992, CAAE: 32371514.0.0000.0071.

## RESULTS

The study included 20 patients admitted to the intensive treatment unit of a private hospital in São Paulo, from January 2012 to December 2013, and diagnosed as bronchospasm and/or wheezing. Age range of 1 to 158 months, median of 16 months (interquartile interval – IQI: 10 to 74 months). The mean length of stay was 5.7 days (standard deviation − SD – of 3.19). Drug treatment was not changed because of the study. Bronchodilators (salbutamol) were used in 100% of cases, oral corticosteroids (prednisolone) were given on 14 (70%) occasions, and 4 (20%) patients received intravenous corticosteroids (methylprednisolone). Inhalation with a hypertonic solution was prescribed for 30% of patients and antibiotics for 80%. The treatment considered prophylactic was maintained during hospitalization for three patients, in which each one of them received a different type of medication, namely: montelukast (Singulair^®^), fluticasone propionate (Flixotide^®^), and fluticasone associated with salmeterol (Seretide^®^). Respiratory physical therapy was done in 100% of patients. The time between the start of conventional treatment and the introduction of Heliox^®^ showed a median of 10 hours (3 to 53 hours).

The variables to evaluate the clinical outcome are shown on table 2 . In two cases, treatment with Heliox^®^ was associated with noninvasive ventilation. Heliox^®^ was discontinued due to treatment failure in three (25%) patients − two of them after 30 minutes and one after 60 minutes. Of these, one required invasive mechanical ventilation.

**Table 2:** Severity score, heart and respiratory rates at zero, 30, 60 and 120 minutes

	Time point (minutes)				p value		
	
	Pre	30	60	120	30	60	120
Score	5.6 (2.0)	4.1 (2.1)	3.4 (1.9)	2.9 (1.5)	<0.001	<0.001	<0.001
HR	152 (25.0)	141 (21.3)	141 (20.3)	138 (18.3)	0.011	0.33	0.12
RR	54 (13.4)	44 (11.3)	38 (10.3)	37 (10.3)	<0.001	<0.001	<0.001

Results expressed as mean (standard deviation).

HR: heart rate; RR: respiratory rate.

**Figure 2:**
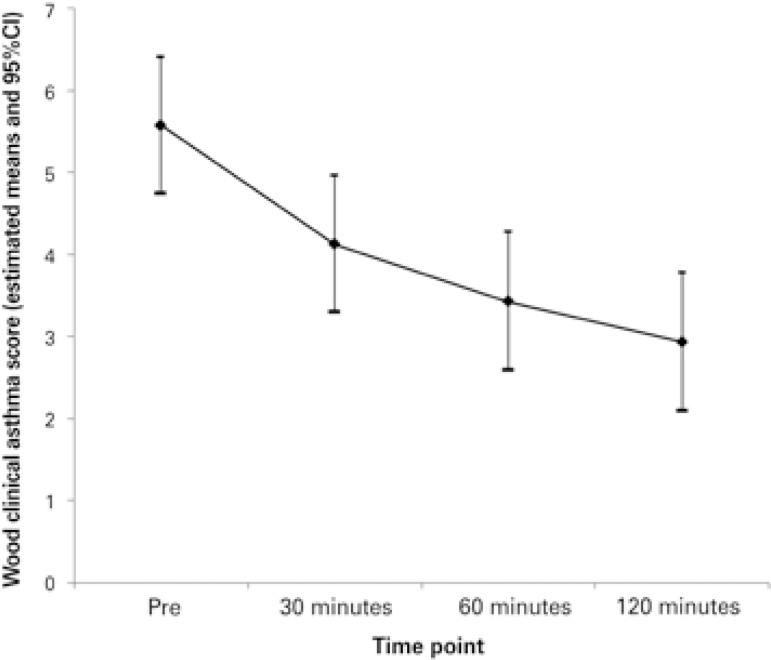
Mean profiles of Wood clinical asthma score per time (pre, 30, 60 and 120 minutes)

**Figure 3:**
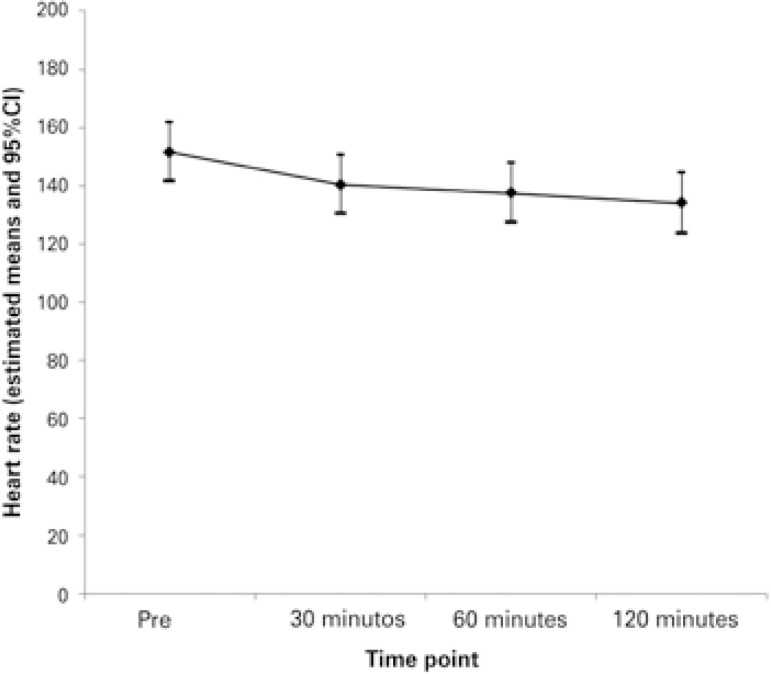
Mean profiles of heart rate per time (pre, 30, 60 and 120 minutes)

**Figure 4:**
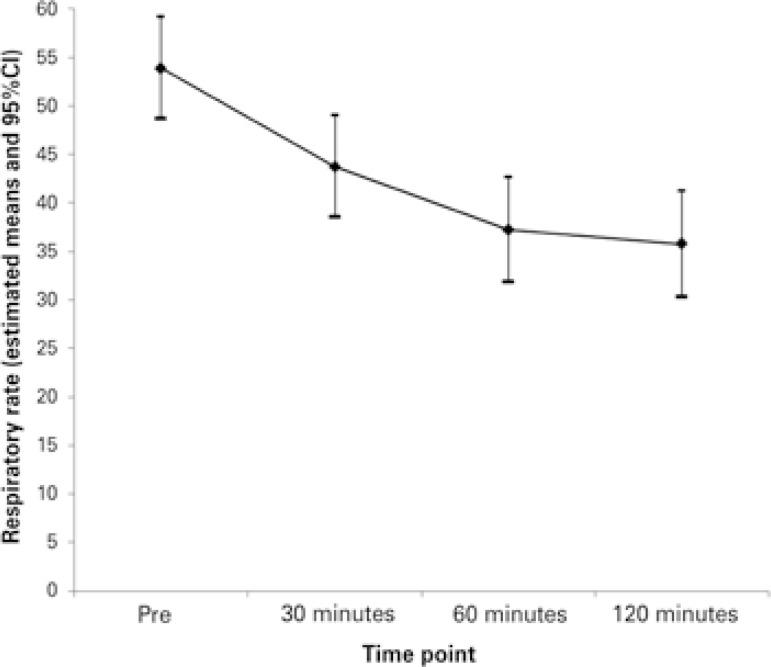
Mean profiles of respiratory rate per time (pre, 30, 60 and 120 minutes)

The mean disease severity score at time point zero was 5.6 (SD 2.0) and at time point 120 minutes, it was 3.4 (SD 2.0). This difference found in the severity score was statistically different as of 30 minutes (p<0.001), according to what is presented in figure 2 . The heart rate evaluation showed significance only at the first time point of the study, but this relevance was not sustained ( Figure 3 ). The variation of respiratory rate showed a similar progression as the severity score, also showing significance as of 30 minutes ( Figure 4 ). We did not observe a relation between the days of hospitalization and the severity score (r=0.15 and p=0.4).

95%CI: 95% confidence interval.

95%CI: 95% confidence interval.

95%CI: 95% confidence interval.

## DISCUSSION

Over the last decades, the number of studies aiming to confirm the efficacy of the helium-oxygen mixture use in obstructive diseases, such as asthma, bronchiolitis and airway obstruction, has increased.^(^ ^4^ ^,^ ^12^ ^-^ ^15^ ^,^ ^21^ ^,^ ^22^ ^)^ Our study proves to be particularly important since it provides data for the Brazilian population, in which we note a scarcity of results as to clinical applicability of the helium-oxygen mixture, especially regarding the pediatric population.

In a cohort study of the Brazilian population, which included 111 infants diagnosed as acute viral bronchiolitis, the mean length of stay was 9.5 days. In this study, treatment of the illness was conventional and there was no use of the helium-oxygen mixture.^(^ ^23^ ^)^ Whereas in the study conducted with asthmatic children, also without the use of Heliox^®^, the mean length of stay was 6.0 days.^(^ ^24^ ^)^ In our study, the mean length of stay was lower, but it is not yet possible to affirm that these results are associated with the use of Heliox^®^. Liet et al., did not observe a relation between use of the mixture and a reduction in hospitalization time.^(^ ^25^ ^)^

Regarding treatment used during hospitalization, our study showed a high rate of utilization of bronchodilators and oral steroids, a treatment strongly recommended by the Global Initiatives for Asthma.^(^ ^26^ ^)^

Another recommendation of the Global Initiatives for Asthma^(^ ^26^ ^)^ would be the use of the heliox only one hour after starting the conventional drug treatment. In our study, the time between the start of drug treatment and the installation of the helium-oxygen mixture was 10 hours. This datum reinforces the result that the improvement of patients was related to the introduction of the Heliox^®^, and not to the drug treatment effect.

Martinón-Torres et al., followed up non-intubated children with bronchiolitis and concluded that children who received Heliox^®^ presented improved respiratory and heart rates, besides remaining less time at the intensive care unit.^(^ ^27^ ^)^ In the children included in our study, besides the distress score, we followed up respiratory and heart rates. Use of the helium-oxygen mixture showed a significant reduction in the respiratory distress score and in respiratory rate of our patients in the first 30 minutes, with a continued reduction for up to 120 minutes.

The most significant improvement of the distress score in the first 30 minutes can be attributed to the change in airflow pattern, which becomes more laminar with the use of Heliox^®^. However, the progressive improvement of the children throughout time may be related to a better lung deposition of the inhaled drug (bronchodilators) with the use of Heliox^®^.

These data confirm the results of Kress et al., who conducted a randomized study with 45 asthmatic children, in which 22 received albuterol with Heliox^®^, and 23 with oxygen. They observed a statistically significant improvement in the forced expiratory volume in one second (FEV1) in the Heliox^®^ group.^(^ ^28^ ^)^ Braun Filho et al., compared the administration of bronchodilators in the conventional manner with the administration of inhaled medication with a mixture of helium gas and observed that the use of Heliox^®^ decreased the risk of permanence of the patient in the observation room.^(^ ^29^ ^)^

In our study, three patients did not present with an improved distress score, and Heliox^®^ was discontinued: one of them used invasive mechanical ventilation. In two successful cases, there was an association with noninvasive ventilation. With a good response, this association is indicated, since Heliox^®^ and noninvasive mechanical ventilation are considered synergistic and complementary therapies; we further point out, however, that the need for ventilators prepared to receive the mixture limits the process. Martinón-Torres et al., conducted a randomized crossover study in 12 infants diagnosed with bronchiolitis, noting a significant improvement of SpO_2_ and of respiratory work, when noninvasive mechanical ventilation was associated with Heliox^®^.^(^ ^30^ ^)^ A review as to the use of noninvasive mechanical ventilation with Heliox^®^ reinforces the complementary role of both therapies, exposing that the association of the two can guarantee a promising future for the treatment of various illnesses.^(^ ^19^ ^)^

Recent studies have demonstrated that the use of Heliox^®^ with a high-flow nasal cannula had a significant influence on the improvement of oxygenation and respiratory rate, and can be a successful alternative in the future, since it demands more accessible equipment than the microprocessed ventilators used in noninvasive mechanical ventilation.^(^ ^31^ ^,^ ^32^ ^)^

In our study, we chose to observe the cases of bronchospasm and wheezing, disorders in which Heliox^®^ improved gas exchange, decreased respiratory work, and prevented reintubation.^(^ ^33^ ^)^

Our protocol included children who maintained the moderate to severe severity score after one hour of conventional treatment. This limited the number of children included in the study, since the majority of children with obstructive diseases improved with conventional intervention during the first hour. Another limitation of the study is that it was retrospective, which limits the results and its interpretations.

There still is no consensus as to the use of the helium-oxygen mixture, and the great difficulty in conclusion is in the difference of study designs, inclusion criteria, disease severity, treatment, and time when intervention started.^(^ ^34^ ^)^

## CONCLUSION

Use of Heliox^®^ in children with obstructive diseases showed positive outcomes, with a significant reduction in the distress score and respiratory rate in the first 30 minutes of use. Therefore, Heliox^®^ can be considered a support in the treatment of patients with respiratory failure from obstructive causes.
